# Supplementary material for: Inadequate Intake of Choline and Essential Fatty Acids in Latin American Childbearing-Age Women as a Regional Pre-Conceptional Disadvantage: ELANS Results
Source: Nutrients. 2024 Sep 18;16(18):3150. doi: 10.3390/nu16183150 (PMC11434761; doi:10.3390/nu16183150)
Supplement: Supplementary file 1 [file nutrients-16-03150-s001.zip › nutrients-3165251-supplementary.pdf]

Inadequate Intake of Choline and Essential Fatty Acids in Latin American Childbearing-Age Women as a Regional Pre-Conceptional Disadvantage: ELANS Results

Marianella Herrera-Cuenca, Martha Cecilia Yépez García, Lilia Yadira Cortés Sanabria, Pablo Hernandez, Guillermo Ramírez, Maura Vásquez, Yaritza Sifontes, Georgina Gómez, María Reyna Liria-Domínguez, Attilio Rigotti, Mauro Fisberg, Irina Kovalskys, and Maritza Landaeta-Jiménez

Supplementary material

Table S1. Mean of the w6/w3 ratio of childbearing-age women aged 15–49 years by country.

Country	n	Mean	Standard Error	95% CI	
				Lower	Upper
ELANS	3704	10,18	0,08	10,02	10,34
Argentina	521	17,94	0,29	17,38	18,50
Ecuador	324	12,84	0,28	12,29	13,39
Chile	345	11,19	0,23	10,74	11,64
Costa Rica	309	9,43	0,12	9,19	9,67
Brazil, Peru, Colombia	1742	8,12	0,04	8,04	8,20
Venezuela	463	7,08	0,05	6,98	7,18
